# 

**Published:** 1874-11

**Authors:** 


☞ This issue of the RECORD has been delayed a few days, owing to the press of business occasioned by the State Fair, the grandest success in that line known to the history of Georgia.

*To Our Subscribers.*--Our thanks are especially due those of our subscribers who have remitted their dues, and thus aided us in carrying out our contract with our publishers. Those who have not done us this favor, will please do so immediately after receiving this number, and thereby relieve us during the "hard times!" Don't fail, friends. Should you not respond within ten days, we will be compelled to forward bills for collection, with collection fees added.

☞ Write your Names, Post Office, County and State *plainly*. Be sure to say what Post Office you wish the RECORD sent, and always date your letters. We receive many well written letters, but doubt even the authors being able to decipher their signatures.

☞ Address all Communications, to POWELL & GOLDSMITH, none other.

☞ Send Money by Check, Postal Order or by Registered Letters. We are willing to endorse for the honesty of our subscribers, but cannot be responsible for the irregularity of the mails.

### LIST OF CASH PAYMENTS TO THE RECORD:

### For 1873.

| | |
|---|---|
| Dr E W Lane, Ga | $2 50 |
| Dr Eugene Grissom, N. C | 2 50 |
| Dr H J Smith, Ga | 2 50 |
| Dr James M Green, Ga | 2 50 |
| Dr W S Kenuall, Ga | 1 25 |
| Dr H J Holmes, Miss | 2 50 |
| Dr R P Means, Ala | 2 50 |
| Dr James Tyson, Philadelphia, Pa | 2 50 |
| Dr A Williamson, Ala | 2 50 |
| Dr J McHenderson | 2 50 |

### For 1874.

| | |
|---|---|
| Dr R E Nelson, Va | 2 50 |
| Dr P D Laster, N. C | 2 50 |
| Dr E W Lane, Ga. | 2 50 |
| Dr J G McCrary, Ga | 2 50 |
| Dr J H Shelton, N. C | 2 50 |
| Dr J J Horton, S. C | 2 50 |
| Dr E Carter, Miss | 2 50 |
| Dr A A Hill. N. C | 2 50 |
| Dr E T Easley, Texas | 2 50 |
| Dr William White, Va | 2 50 |
| Dr E D Mann, N. C. | 2 50 |
| Dr C B Slaughter, Miss | 2 50 |
| Medical College of Ga | 2 50 |
| Dr Robert Kells, Miss | 2 50 |
| Dr Eugene Grissom, N. C | 2 50 |
| Dr W T Mathes, Tenn | 2 50 |
| Dr H J Smith, Ga | 2 50 |
| Dr J M Runyan, Indiana | 2 50 |
| Dr P W Callihan, La | 2 50 |
| Dr W M Compton, Miss | 2 50 |
| Dr F M Gray, Ala | 2 50 |
| Dr J Harris, Ga | 2 50 |
| Dr W S Kendall, La | 2 50 |
| Dr James M Green, Ga | 2 50 |
| Dr E P Gains, Ala | 2 50 |
| Drs Lanier & McCabe, Miss | 2 50 |
| Dr A M Dantzler, Ga | 2 50 |
| Dr B J Foster, Ala | 2 50 |
| Dr James Ray, La. | 2 50 |
| Dr J Paul Cheney, Ga | 2 50 |
| Dr J B Manson, Ga | 2 50 |
| Dr J F Pow, Miss | 2 50 |
| Dr D M McRae, Miss | 2 50 |
| Drs Moody & Bevin, Ala | 2 50 |
| Dr A Brock, Arkansas | 2 50 |
| Dr L R Lincecum, Texas | 2 50 |
| Dr J L Gibson, Ga | 2 50 |
| Dr J W Rickman, Ala | 2 50 |
| Dr E D Yeates, Miss | 2 50 |
| Dr J S Hughson, S. C | 2 50 |
| Dr W M Fitch, S. C. | 2 50 |
| Dr J S Horsley, Ga | 2 50 |

### For 1874.

| | |
|---|---|
| Dr J J Grace, Ala | $2 50 |
| Dr J J Mann, N. C | 2 50 |
| Dr H J Holmes, Miss | 2 50 |
| Dr William Walker, La | 2 50 |
| Dr Louis Hadden, La. | 2 50 |
| Dr H T Chevis, La | 2 50 |
| Dr H P Guilbean, La | 2 50 |
| Dr J S Beard, Ala | 2 50 |
| Dr G W Tribble, Miss | 2 50 |
| Dr E Y Fleming, Miss | 2 50 |
| Dr G D Norris, Ala | 2 50 |
| Dr Samuel J Alexander, N. C | 2 50 |
| Dr B F Nicholds, Ala | 2 50 |
| Dr A H Sellers, Ala | 2 50 |
| Dr LaFayette Garrett, Texas | 2 50 |
| Dr H McNeill, S. C | 2 50 |
| Dr L M Dampeer, Miss | 2 50 |
| Dr A S Campbell, Miss | 2 50 |
| Dr W C McMillen, S. C | 2 50 |
| Dr W S Morgan, Ga | 2 50 |
| Dr A Reskitt, Ga | 2 50 |
| Dr G A Whitney, Oregon | 2 50 |
| Dr J G W Brown, Ga | 2 50 |
| Dr B E Reese, Va | 2 50 |
| Dr R F Hall, Ga | 2 50 |
| Dr L D Johnson, Ga | 2 50 |
| Dr C G Stovall, Ala | 2 50 |
| Dr R P Means, Ala | 2 50 |
| Dr T C Morton, Ala | 2 50 |
| Dr L E Kirkman, N. C | 2 50 |
| Dr W J Reese, Ga | 2 50 |
| Dr W F Wilson, Ga | 2 50 |
| Dr J B Fuller, Ala | 2 50 |
| Dr J F Wootten, Ga | 2 50 |
| Dr Thomas H Edwards, Fla | 2 50 |
| Dr James Tyson, Philadelphia, Pa | 2 50 |
| Dr A M Winn, Ga | 2 50 |
| Dr Z J Scott, Miss | 2 50 |
| Dr Geo A Dyer, Indiana | 2 50 |
| Dr W A Culbertson, Ga | 2 50 |
| Dr E B Rees, Ga | 2 50 |
| Dr A Kindman, Ga | 2 50 |
| Dr William T Downy, Ala | 2 50 |
| Dr R H Day, La | 2 50 |
| Dr A G Symthe, Miss | 2 50 |
| Dr A Williamson, Ala | 2 50 |
| Dr W S Selman, Ga | 2 50 |
| Dr B F Foy, N. C | 2 50 |
| Dr O P Langworthy, La | 2 50 |
| Dr S B Pearce, Ga | 2 50 |
| Dr J N Taylor, La | 2 50 |
| Dr J McHenderson | 2 50 |
| Dr R D Jackson, Ala | 2 50 |

**The above list of names are those who have paid for volumes 3 and 4. If we have failed to credit any, they will do us the favor to inform us of the fact.**

### ASSOCIATE EDITORS:

**GEORGIA.**

James A. Long, M.D.
W. L. M. Harris, M.D.
H. L Battle, M.D.
W. C. Musgrove, M D
L. B. Bouchelle, M.D.
Thomas Burdell, M.D.
E. H. W. Hunter, M.D.
V. S Hitt, M.D.
J. E. Pope, M.D.
A. H. Brantley, M.D.
J. J. Knott, M.D.
J. C. Wisdom, M.D.
W. W. Leake, M.D.
S. G. WHITE, M.D.
W. H. HALL, M.D.
G. D. CASE, M.D.

**KENTUCKY.**

W. S. Taylor, M.D.
B. W. Stone, M.D.
Charles Kearns, M.D.

**TEXAS.**

S. M. Welch, M.D.
T. J. Heard, M.D.
W. A. Heard, M.D.

**TENNESSEE.**

J. D. Tucker, M.D.

**ALABAMA.**

J. C. Knox, M.D.
Geo. F. Taylor, M.D.
J. B. Barnett, M.D.
S. M. Hogan, M.D.
D. S. Hopping, M.D.
J. T. Searcy, M.D.
S. F. Hill, M.D.

**NORTH CAROLINA.**

J. B. Hughes, M.D.
S. S. Satchwell, M.D.
A. B. Pierce, M.D.
H. Kelly, M.D.
Geo. A. Foote, M.D.
E. A. Anderson, M.D.
H. T. Bahnson, M.D.
Willis Alston, M.D.
Thos. F. Wood, M.D.
N. J. Pittman, M.D.
John W. Booth, M.D.

**SOUTH CAROLINA.**

E. T. McSwain, M.D.
G. M. Rivers, M.D.

**OHIO.**

J. Q. A. Hudson, M.D.
C. H. Tidd, M.D.

**VIRGINIA.**

John H. Claiborne, M.D.
F. Horner, Jr., M.D.
R. J. Preston, M.D.
J. E. Lockridge, M.D.
J. S. Wharton, M.D.
Wm. O. Owen, M.D.
Sam'l P. Ripley, M.D.
Benj. Blackford, M.D.
E. A. Drewry, M.D.
Rob B. Tunstall, M.D.
W. F. Barr, M.D.
L. B. Anderson, M.D.
J. M. Lazzell, M.D.

**MISSISSIPPI.**

C. B. Gallaway, M.D.
Edward Lea, M.D.
S. V. D. Hill, M.D.
E. T. Henry, M.D.
T. P. Lockwood, M.D.
Robert Kells, M.D.

**ARKANSAS.**

A. D. Hodge, M.D.
J. K. Hodge, M.D.
A. W. Hobson, M.D.

**NEW JERSEY.**

J. B. MATTISON, M.D.

### CORRESPONDING EDITORS:

Ely Van DeWarker, M. D., New York.
C. C. F. Gay, M.D., New York,
*Surgeon of Buffalo General Hospital.*
M. H. Henry, M.D., New York,
*Surgeon to N. Y. Dispensary, Dep't of Venerial and Skin Diseases.*
Benjamin Howard, M.D., New York.
James Tyson, M.D., Philadelphia, Pa.
W. W. Keen, M.D. Philadelphia.
Robert Robson, M.D., Indiana.
A. Patton, M.D., Indiana.
John H. Weir, M.D., Illinois.
Thomas J. Gallaher, M. D, Pennsylvania.
S. C. Busey, M.D., Washington, D. C.
Wm. R. Whitehead, M.D., Colorado Territory.

☞ The RECORD will be mailed between the 20th and 25th of eac[illegible] month.

